# Green Synthesized Silver Nanoparticles: Antibacterial and Anticancer Activities, Biocompatibility, and Analyses of Surface-Attached Proteins

**DOI:** 10.3389/fmicb.2021.632505

**Published:** 2021-04-22

**Authors:** Magdalena Wypij, Tomasz Jędrzejewski, Joanna Trzcińska-Wencel, Maciej Ostrowski, Mahendra Rai, Patrycja Golińska

**Affiliations:** ^1^Department of Microbiology, Nicolaus Copernicus University, Toruń, Poland; ^2^Department of Immunology, Nicolaus Copernicus University, Toruń, Poland; ^3^Department of Biochemistry, Nicolaus Copernicus University, Toruń, Poland; ^4^Nanobiotechnology Laboratory, Department of Biotechnology, SGB Amravati University, Amravati, India

**Keywords:** biogenic AgNPs, protein molecules, capping agents, antibacterial agent, anticancer agent, cytotoxicity, MCF-7, RAW 264.7

## Abstract

The increasing number of multi-drug-resistant bacteria and cancer cases, that are a real threat to humankind, forces research world to develop new weapons to deal with it. Biogenic silver nanoparticles (AgNPs) are considered as a solution to this problem. Biosynthesis of AgNPs is regarded as a green, eco-friendly, low-priced process that provides small and biocompatible nanostructures with antimicrobial and anticancer activities and potential application in medicine. The biocompatibility of these nanoparticles is related to the coating with biomolecules of natural origin. The synthesis of AgNPs from actinobacterial strain was confirmed using UV-Vis spectroscopy while their morphology, crystalline structure, stability, and coating were characterized using, transmission electron microscopy (TEM), X-ray diffraction (XRD), Zeta potential and Fourier transform infrared spectroscopy (FTIR). Antibacterial activity of biogenic AgNPs was evaluated by determination of minimum inhibitory and minimum biocidal concentrations (MIC and MBC) against *Escherichia coli*, *Klebsiella pneumoniae*, *Pseudomonas aeruginosa*, and *Staphylococcus aureus*. The potential mechanism of antibacterial action of AgNPs was determined by measurement of ATP level. Since the use of AgNPs in biomedical applications depend on their safety, the *in vitro* cytotoxicity of biosynthesized AgNPs on MCF-7 human breast cancer cell line and murine macrophage cell line RAW 264.7 using MTT [3-(4,5-dimethylthiazol-2-yl)-2,5-diphenyltetrazolium bromide] assay, cell lactate dehydrogenase (LDH) release and measurement of reactive oxygen species (ROS) level were assessed. The nanoparticle protein capping agent that can be involved in reduction of silver ions to AgNPs and their stabilization was identified using LC-MS/MS. Nanoparticles were spherical in shape, small in size (mean 13.2 nm), showed crystalline nature, good stability (−18.7 mV) and presence of capping agents. They exhibited antibacterial activity (MIC of 8–128 μg ml^−1^, MBC of 64–256 μg ml^−1^) and significantly decreased ATP levels in bacterial cells after treatment with different concentrations of AgNPs. The *in vitro* analysis showed that the AgNPs demonstrated dose-dependent cytotoxicity against RAW 264.7 macrophages and MCF-7 breast cancer cells but higher against the latter than the former. Cell viability decrease was found to be 42.2–14.2 and 38.0–15.5% while LDH leakage 14.6–42.7% and 19.0–45.0%, respectively. IC_50_ values calculated for MTT assay was found to be 16.3 and 12.0 μg ml^−1^ and for LDH assay 102.3 and 76.2 μg ml^−1^, respectively. Moreover, MCF-7 cells released a greater amount of ROS than RAW 264.7 macrophages during stimulation with all tested concentrations of AgNPs (1.47–3.13 and 1.02–2.58 fold increase, respectively). The SDS-PAGE (sodium dodecyl sulfate-polyacrylamide gel electrophoresis) analysis revealed the presence of five protein bands at a molecular weight between 31.7 and 280.9 kDa. These proteins showed the highest homology to hypothetical proteins and porins from *E. coli*, *Delftia* sp. and *Pseudomonas rhodesiae*. Based on obtained results it can be concluded that biogenic AgNPs were capped with proteins and demonstrated potential as antimicrobial and anticancer agent.

## Introduction

In recent years, increasing bacterial resistance to available antibiotics has generated the need to search for alternative approaches to combat microorganisms causing serious or even life-threatening infections (Jindal et al., 2015; Yuan et al., 2017). Similarly, cancer causes leading mortality among other mankind diseases (Jeyaraj et al., 2013). In this context, novel agents with antimicrobial and anticancer activities, such as metallic nanoparticles (MNPs), have attracted the considerable attention of both academia and industry. MNPs, mainly silver nanoparticles (AgNPs), with effective functionalities have gained particular attention in the biomedical and other health-related areas of the modern world (Jia et al., 2008; Gurunathan et al., 2014; Firdhouse and Lalitha, 2015; Bilal et al., 2017). They have been used for the production of disinfectants, in dental composites or as bactericidal coatings (Rai et al., 2018). AgNPs engineered by green routes using bacteria, actinomycetes, fungi, algae, and plants offer many advantages compared with those synthesized by physical and chemical approaches (Rai et al., 2015; Qayyum et al., 2017; Oves et al., 2018, 2019). Their synthesis is easy, cheap, and eco-friendly, and the nanoparticles thus obtained are coated with capping agents of natural origin that allows for elimination of post-synthetic coating step needed in case of chemical synthesis which provides stable nanostructures required for medical applications (El-Nour et al., 2010; Khandel et al., 2018; Fahmy et al., 2019). In the extracellular synthesis of AgNPs, silver ions may be reduced by biomolecules such as amino acids, proteins, NAD(P)+ reductases, dehydrogenases, and various secondary metabolites (Xu et al., 2020). Their capping agents are formed by extracellular proteins, enzymes, or peptides (Akhtar et al., 2013; Karatoprak et al., 2017). The capping agents are adsorbed onto the surface of biosilver nanoparticles and act as stabilizing agent that protects from agglomeration and affect the morphology of nanoparticles by preventing their uncontrolled growth (Saranyaadevi et al., 2014; Gulati et al., 2018). It is also claimed that biogenic nanoparticles coated with biomolecules display higher biocompatibility in nature (Mohanpuria et al., 2008; Singh et al., 2018). AgNP capping agents enable various surface modifications and attachments of other compounds that may be utilized for various medical applications (Mohanpuria et al., 2008; Gurunathan et al., 2014; Firdhouse and Lalitha, 2015). In this context, although biogenic AgNPs themselves display a broad range of antimicrobial activities against pathogenic bacteria and fungi (Augustine and Rajarathinam, 2012; Anasane et al., 2016; Wypij et al., 2016, 2018a) they can be functionalized by other compounds, e.g., antibiotics (ampicillin, kanamycin, tetracycline, etc.) and antifungal agents (amphotericin B, fluconazole, ketoconazole, etc.) to obtain nanoconstructs with enhanced biological activity against various microorganisms (e.g., *Escherichia coli*, *Klebsiella pneumoniae*, *Staphylococcus aureus*, *Bacillus subtilis*, and *Candida albicans*) when compared to compounds used separately (Wypij et al., 2018a). Unfortunately, the knowledge on the structure of the capping agent and the exact role of such biomolecules in the stabilization of AgNPs is still scarce.

Human exposure to nanoparticles is inevitable as they are being widely used, and therefore, the emerging toxicity issues are now gaining attention. For biomedical purposes, especially *in vivo* applications, toxicity is a critical factor to consider when evaluating their potential (Lewinski et al., 2008). Exposure to certain cytotoxic agents can disturb the cell membrane, which allows cellular contents to leak out or affect mitochondrial activity (Lewinski et al., 2008). It is also claimed that the induction of reactive oxygen species (ROS) is a general mechanism of NP-mediated cytotoxicity. An *in vitro* exposure to AgNPs cause reduction of glutathione (GSH) level, elevated ROS levels, lipid peroxidation, and increased expression of ROS responsive genes. An increase in ROS level was reported to be associated with DNA damage, apoptosis, and necrosis (Arora et al., 2008; Carlson et al., 2008; Kim et al., 2009). It is interesting that AgNPs exhibit anti-cancer properties against various cancer cell lines such as MCF-7 breast cancer cells (Gurunathan et al., 2013; Jeyaraj et al., 2013; Gajendran et al., 2014; Kwan et al., 2016; Oves et al., 2018), HCT116 colon cancer cells (Gurunathan et al., 2018), prostate cancer cells (Firdhouse and Lalitha, 2013), HeLa cells (Pandian et al., 2019), lung carcinoma A549 cells (Gurunathan et al., 2015), etc. The present work is a continuation of our studies on the biological synthesis of AgNPs using the actinobacteria, *Pilimelia columellifera* subsp. *pallida* strain SF23, which was isolated from pine forest soil (Anasane et al., 2016). The biogenic AgNPs thus obtained were characterized by Fourier-transform infrared (FTIR) spectroscopy that revealed the presence of functional groups associated with proteins or peptides on their surface. Moreover, the biosynthesized AgNPs were screened against fungi causing superficial mycoses and showed antifungal activity against *C. albicans*, *Candida tropicalis*, *Malassezia furfur*, and *Trichophyton rubrum* with the highest activity against the latter. These results revealed the biosynthesized nanoparticles can be considered as an antifungal agent (Anasane et al., 2016).

In this study, the toxic effect of AgNPs biosynthesized from strain SF23 was evaluated against selected Gram-positive and Gram-negative bacteria based on the determination of ATP level in their cells, as well as against MCF-7 human breast cancer cell line and murine macrophage cell line RAW 264.7 by 3-(4,5-dimethylthiazol-2-yl)-2,5-diphenyltetrazolium bromide (MTT) assay, measurement of lactate dehydrogenase (LDH) release and determination of ROS level using DCF-DA method. Moreover, the capping proteins of biogenic nanoparticles were identified by LC-MS/MS.

## Materials and Methods

### Nanoparticle Biosynthesis

Actinobacterial strain SF23 was grown in yeast extract-malt extract broth (ISP2; International *Streptomyces* Project No. 2 medium) pH 5.5 (Shirling and Gottlieb, 1966), for 7 days at 27°C. The actinobacterial biomass was centrifuged at 6,000 × g for 10 min, washed three times with sterile distilled water and autolyzed by re-suspending in sterile distilled water at 27°C for 3 days. The cell debris was centrifuged at 6,000 × g for 15 min and separated supernatant mixed with silver nitrate (AgNO_3_; final concentration 0.001 mol l^-1^). The sample was incubated for 3 days at 25°C in the darkness for the synthesis of nanoparticles.

### Characterization of Biosynthesized AgNPs

#### Visual and UV-Spectroscopic Analysis

The synthesis of AgNPs was observed visually by the color change of the autolysat with AgNO_3_ from colorless to brown and spectroscopically using ultraviolet-visible (UV) spectroscopy (NanoDrop ND2000, Thermo Scientific, Waltham, MA, United States) in a wavelength range from 200 to 800 nm, at a resolution of 1 nm. The autolysate without silver nitrate was used as the control sample. Biosynthesized AgNPs were collected by centrifugation at 13,000 × g for 1 h) and dried at 40°C for mass evaluation (mg).

#### Transmission Electron Microscopy Analysis

The size and morphology of the AgNPs were analyzed by TEM (FEI Tecnai F20 X-Twintool, Fei, Hillsboro, OR, United States), operating at an acceleration voltage of 100 kV after applying the AgNPs re-suspended in molecular grade sterile water on a carbon-coated copper grid (400 μm mesh size) and drying at room temperature. The obtained data were analyzed by Statistica Software (StatSoft Inc., Tulsa, OK, United States).

#### X-Ray Diffraction Analysis

X-ray diffraction analysis was conducted using X-ray diffractometer (X’Pert Pro Analytical Phillips, Lelyweg, Netherlands) equipped with Ni filter and Cu k*α* (*λ* = 1.54056 Å) radiation source to determine the crystalline structure of biosynthesized AgNPs. The powder of AgNPs was used in this analysis. The diffraction pattern was recorded over a 2θ range of 5°–120°. The obtained diffractogram was compared to the standard database of the International Centre for Diffraction Data (ICDD).

#### Zeta Potential Analysis

The zeta potential measurement was performed to estimate the surface charge of biosynthesized AgNPs, which reflects their stability. The colloidal suspension of AgNPs was 10-fold diluted with molecular grade sterile water and homogenized using ultrasonic homogenizer (Sonic Ruptor 250, Omni Int., Kennesaw, GA, United States) for 15 min at 20 Hz to break down aggregates of nanoparticles. The nanoparticle solution was then filtered through a Millipore filter (0.22 μm) and analyzed using Zetasizer (Malvern Instruments Ltd., Malvern, United Kingdom).

#### Fourier Transform Infrared Spectroscopy Analysis

The analysis of functional groups on the surface of AgNPs was performed using FTIR spectrometer (Spectrum 2000, Perkin-Elmer, Waltham, Massachusetts, United States) in the wavelength range of 4,000–400 cm^−1^. A sample, in the powder form, for FTIR analysis was prepared by combining dried nanoparticles with potassium bromide (KBr).

### Antibacterial Assays

#### Determination of Minimum Inhibitory Concentration and Minimum Biocidal Concentration

The biological activity of AgNPs from strain SF23 was determined against both Gram-negative (*E. coli* ATCC 8739, *K. pneumoniae* ATCC 700603, *Pseudomonas aeruginosa* ATCC 10145) and Gram-positive (*S. aureus* ATCC 6538) bacteria using a standard fold broth microdilution method according to Clinical and Laboratory Standards Institute (CLSI) guidelines (CLSI, 2012). Microorganisms prior to assay to be set were grown in Triptic Soy Broth (TSB; Becton Dickinson) at 37°C for 24 h under shaking conditions at 125 rpm.

Assays were performed (in triplicate) in 96-well plates. The tested concentration range of AgNPs was from 0.016 to 1,024 μg ml^−1^ and the final concentration of bacteria in each well of a plate was 5 × 10^5^ c.f.u (colony forming units) per ml. Both positive (inoculated medium) and negative (non-inoculated medium) controls were also maintained. Inoculated multi-well plates were incubated at 37°C for 24 h. The MIC values were defined as the lowest concentration of AgNPs which inhibited the growth of bacteria that was estimated visually. Subsequently, the MBC values of AgNPs were determined after spreading of 100 μl of each test sample (≥MIC) onto Tryptic Soy Agar (TSA; Becton Dickinson, United States) plates and incubation at 37°C for 24 h. MBC was defined as the lowest concentration of AgNPs that prevented the growth of >99.9% bacterial cells.

#### Quantitative Determination of ATP in Bacterial Cells Treated With AgNPs

The quantitative determination of ATP in the bacterial cells, as given above, after treatment with AgNPs (concentration range 0.125–512 μg/ml) was determined using BacTiter-Glo™ kit (Promega, Madison, WI, United States) following the manufacturer’s instruction. The test was performed in triplicate, in sterile 96-well plates. Each well contained sterile distilled water as a diluent, glucose (0.2% final concentration), AgNPs at the specified final concentration, as indicated above, and inoculum of bacteria (1.2 × 10^7^ CFU/ml). The total volume of the test sample in each well was 100 μl. The appropriate controls were also maintained as follows:

- positive, containing glucose (0.2%) solution and test microorganism,
- negative, containing glucose (0.2%) solution,
- background, which was the appropriate concentration of AgNPs and glucose (0.2%) solution.

Plates were incubated for 4 h at 37°C. Subsequently, the test samples (50 μl) were transferred to 96-well opaque plates and mixed with buffer containing substrate (luciferin, BacTiter-Glo™, Promega, Madison, WI, United States) and thermostable luciferase (Ultra-Glo™ Recombinant Lucyferase, Promega, Madison, WI, United States) in a ratio of 1:1 (v/v). Plates were incubated for 5 min at room temperature and luminescence of samples measured at 600 nm by using Synergy HT BioTek Reader (United States). The results were expressed as the percent of inhibition of ATP synthesis in bacterial cells treated with AgNPs when compared with control (untreated cells).

### Cytotoxicity Assays

#### Cell Culture

Cytotoxic assays were performed using MCF-7 human breast cancer cell line (Lot. 13 K023) and RAW 264.7 cells (murine macrophage cell line; cat. no. 91062702) purchased from the European Collection of Cell Cultures (ECACC; Salisbury, United Kingdom). MCF-7 cells were maintained in RPMI 1640 medium containing 2 mM L-glutamine and supplemented with heat-inactivated 10% fetal bovine serum (FBS), 100 μg ml^−1^ streptomycin, and 100 IU ml^−1^ penicillin, and non-essential amino acids. RAW 264.7 cells were cultured in Dulbecco’s modified Eagle’s medium (DMEM) supplemented with 10% FBS, 100 μg ml^−1^ streptomycin, and 100 IU ml^−1^ penicillin. Both cell lines were cultured at 37°C in a humidified atmosphere of 5% CO_2_ and the macrophages were subjected to no more than 15 cell passages. All reagents for cell culture were purchased from Sigma-Aldrich (Darmstadt, Germany).

#### MTT Assay for Testing Cell Viability

Cell viability, after treatment with different concentrations of AgNPs, was assessed using MTT assay. This colorimetric test, which is based on the reduction of yellow tetrazolium salt [3-(4,5-Dimethyl-2-thiazolyl]-2,5-diphenyl-2H-tetrazolium bromide; MTT; Sigma Aldrich, Darmstadt, Germany) to blue formazan product by mitochondrial dehydrogenase in the metabolically active cells, reflects the metabolic rate of treated cells. MCF-7 cells and RAW 264.7 macrophages were seeded at a density of 1 × 10^4^ per well into 96-well plates in appropriate culture medium and pre-incubated for 24 h at 37°C humidified atmosphere containing 5% CO_2_. The cells were stimulated for 24 h with different concentrations of AgNPs (1, 2, 3, 4, 5, 10, 16, 32, and 64 μg ml^−1^) in the respective cell culture medium. Aliquots of 10 μl of filtered (0.22 μm) MTT (0.5 mg ml^−1^) dissolved in PBS were then added into wells and the plates incubated in the darkness and humidified atmosphere containing 5% CO_2_ for 4 h at 37°C. The supernatants were then removed and 100 μl of dimethyl sulfoxide was added. The plates were mixed horizontally for 15 min and the optical density was measured at 570 nm (with a reference wavelength of 630 nm) using Synergy HT Multi-Mode Microplate Reader (BioTek; Winooski, VT, United States). The blank wells contained the respective culture medium with the corresponding AgNPs concentrations. The results were expressed as a percentage of control untreated cells which served as 100%.

The percentage of cell viability was computed according to the following formula:

$$\%\mathrm{cell viability}=\left[\left(\mathrm{At}-\mathrm{Abt}\right)/\left(\mathrm{Ac}-\mathrm{Abc}\right)\right]\times 100.$$

where, At – absorbance value of tested AgNPs; Abt – absorbance value of blank well contained culture medium with the corresponding AgNP concentration; Ac – absorbance value of control cells; Abc – absorbance value of blank well contained culture medium.

All the data were from three independent experiments with six wells for each experiment. The half inhibitory concentrations (IC_50_) of AgNPs were determined using GraphPad Prism 7.0 (GraphPad Software Inc., La Jolla, CA, United States).

#### LDH Assay for Testing Cell Cytotoxicity

The leakage of LDH, a stable cytosolic enzyme, from cells after membrane damage was determined using a CytoTox 96 Non-Radioactive Cytotoxicity Assay (Promega, Madison, WI, United States). In this enzymatic assay, the enzyme released into culture supernatants converse a tetrazolium salt (iodonitrotetrazolium violet; INT) into a red formazan product. The amount of color formed is proportional to the number of lysed cells.

The cells were seeded in a 96-well plate at a density of 1×10^4^ cells per well in 100 μl of the appropriate culture medium for 24 h at 37°C and 5% CO_2_ atmosphere. The medium was replaced with fresh medium containing various concentration of AgNPs (1, 2, 3, 4, 5, 10, 16, 32, and 64 μg ml^−1^). After cell stimulation, 50 μl aliquots of supernatants were transferred to the new wells of 96-well plate and mixed with equal amounts of freshly prepared CytoTox 96 Reagent (Promega, Madison, WI, United States). The microtiter plate was incubated for 30 min at room temperature in the darkness and absorbance measured at 490 nm using the Synergy HT Multi-Mode Microplate Reader (BioTek; Winooski, VT, United States). The AgNP-mediated cytotoxicity was calculated according to the formula:

%cytotoxicity=experimentalLDHrelease/maximumLDHrelease.

Culture media backgrounds containing AgNPs concentration were subtracted from the corresponding wells with cells. Blank wells contained the corresponding AgNPs concentration in culture medium. The positive control wells contained cells treated with 0.8% Triton X-100 solution (provided by the manufacturer) for 45 min. These cells released a maximum amount of LDH, which served as 100%.

All obtained data were from three independent experiments with five wells for each experiment. The half inhibitory concentrations (IC_50_) of AgNPs were determined using GraphPad Prism 7.0 (GraphPad Software Inc., La Jolla, CA, United States).

#### Reactive Oxygen Species Determination Using DCF-DA Method

Intracellular formation of ROS was assessed using oxidation sensitive dye 2',7'-dichlorofluorescein diacetate (DCF-DA; Sigma-Aldrich, Darmstadt, Germany) as a substrate. DCFH-DA is a non-fluorescent compound that is passively diffused into cells where is hydrolyzed by esterases to yield non-permeable DCFH. In the presence of ROS, DCFH is oxidized to the fluorescent DCF. MCF-7 cells or RAW 264.7 macrophages were seeded into 96-well microtiter plates at a density of 25 × 10^3^/well and pre-incubated for 24 h at 37°C. The cells were then stimulated with the different concentrations of AgNPs for 24 h. Subsequently, the cells were washed twice with phosphate-buffered saline (PBS) and incubated in the darkness with 20 μM DCFH-DA (200 μl per well) for 45min at 37°C. The fluorescence was measured using a Synergy HT Multi-Mode Microplate Reader (BioTek; Winooski, VT, United States) with excitation at 485 nm and emission at 528 nm. Culture media backgrounds containing AgNPs concentration were subtracted from the corresponding wells with cells. The ROS level was calculated according to a formula, as given above for the MTT assay, in which the absorbance values were replaced with fluorescence values. All data were from three independent experiments with five wells for each experiment. The results were expressed as the fold change relative to equivalent control untreated cells.

### Identification of AgNP Coating Proteins

#### Sample Preparation

The biomass of strain SF23 after growth in ISP2 broth, as mentioned previously, was washed three times with sterile distilled water, suspended in 50 ml of a 50 mM Tris-HCl buffer (pH 8.0), containing 300 mM NaCl, 10% (v/v) glycerol and 1 mM EDTA and sonicated three times for 20 s using Omni Ruptor sonic homogenizer (Omni Int., GA, United States). The sample was then centrifuged at 10,000 × g for 15 min to separate cell debris. The supernatant was mixed with AgNO_3_, incubated for 72 h in the darkness (as mentioned previously). The sample untreated AgNO_3_ was also analyzed. The Bradford method (Bradford, 1976) was used to determine the total protein concentration in the prepared samples.

#### SDS-PAGE Protein Separation

The SDS-PAGE (electrophoresis under denaturing conditions) was conducted using 10% (w/v) running gel and 4% (w/v) stacking gel, according to method described previously (Ogita and Markert, 1979). The samples containing 20 μg of protein and a standard solution were loaded into polyacrylamide gel and separated at 150 V until the dye reached the end of the separating gel. A Coomassie Brilliant Blue R-250 was used for gel staining (Harlow and Lane, 1988).

#### Identification of Proteins by LC-MS/MS

The separated protein bands were cut from polyacrylamide gel, digested with trypsin and analyzed using nanoAcquity ultra-performance liquid chromatography (UPLC; Waters, Etten-Leur, Netherlands) coupled to an Orbitrap Velos tandem mass spectrometer (Thermo Scientific, Waltham, MA, United States) in the Laboratory of Mass Spectrometry, Institute of Biochemistry and Biophysics Polish Academy of Sciences in Warsaw, Poland. The obtained data were analyzed using the MASCOT program.^1^

### Statistical Analysis

All values are reported as means ± standard error of the means (SEM). Analysis of variance followed by the Bonferroni multiple comparisons test with the level of significance set at *p* < 0.05 were used for statistical analyses performed with GraphPad Prism 7.0 (La Jolla, CA, United States).

## Results

### Characterization of AgNPs Synthesized From Actinobacterial Strain SF23

**Figure 1:**
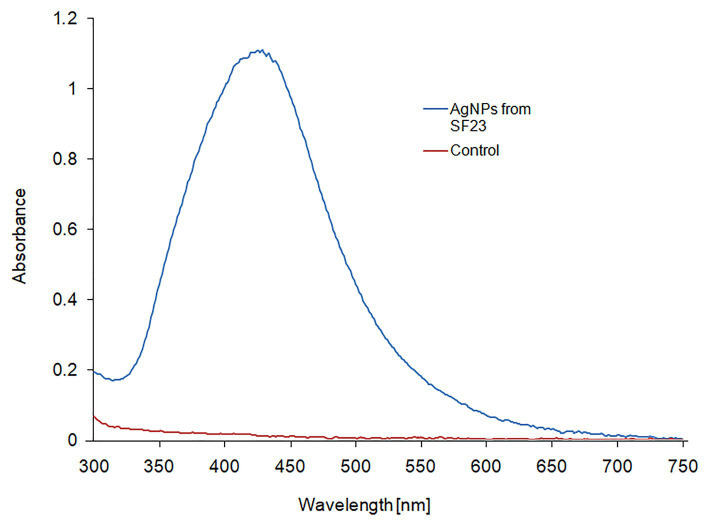
UV-Vis spectrum of biosynthesized AgNPs.

The UV-vis spectroscopic analysis of biosynthesized AgNPs showed a peak with a maximum absorbance at wavelength of 429 nm (Figure 1), which is in the range specified for AgNPs and confirmed their presence in the reaction mixture.

**Figure 2:**
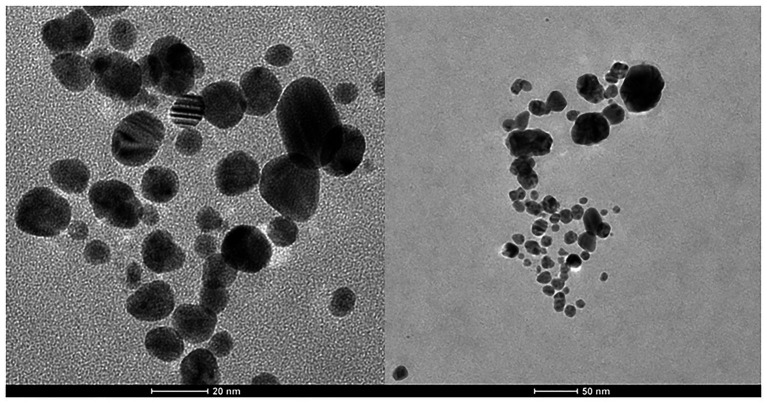
TEM of biosynthesized AgNPs.

Transmission electron microscopy analysis revealed that biosilver nanoparticles were spherical and polydispersed (Figure 2) with a size range of 3–36 nm (mean size = 13.2 nm).

**Figure 3:**
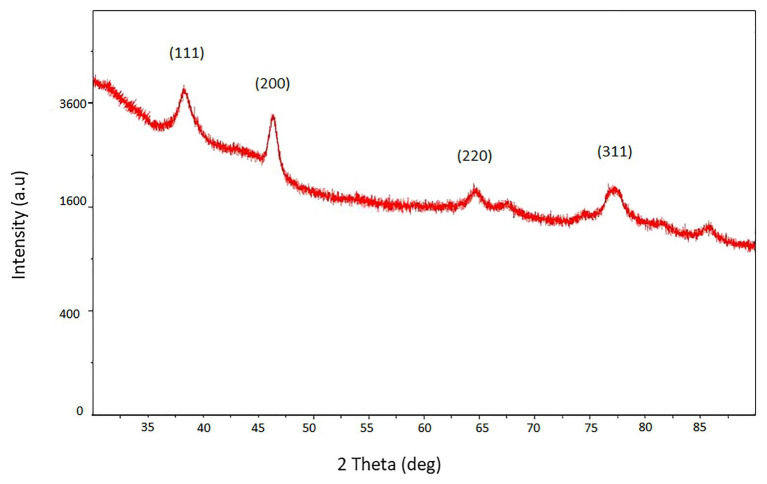
XRD pattern of biosynthesized AgNPs.

The XRD pattern of biosilver nanoparticles was presented in Figure 3. The pattern clearly shows the main peaks at (2θ) = 38.2°, 48.3°, 64.5°, 77.4° corresponding to the (111), (200), (220), and (311) planes, respectively.

**Figure 4:**
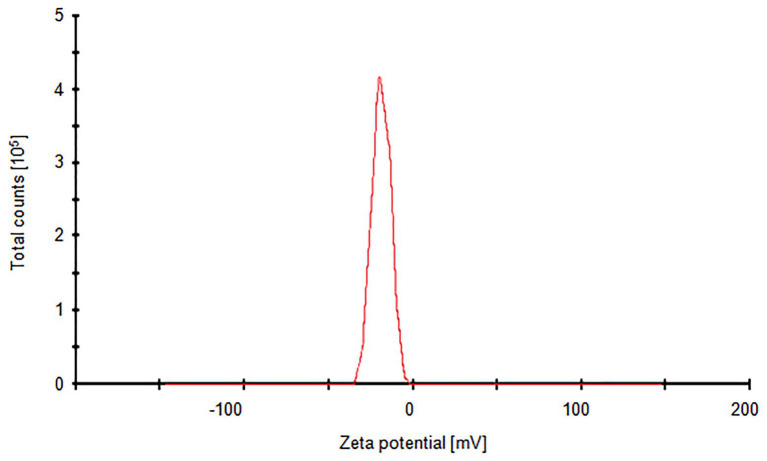
Zeta potential graph of biosynthesized AgNPs.

Silver nanoparticles synthesized from strain SF23 were negatively charged (−18.7 mV), as shown in Figure 4.

**Figure 5:**
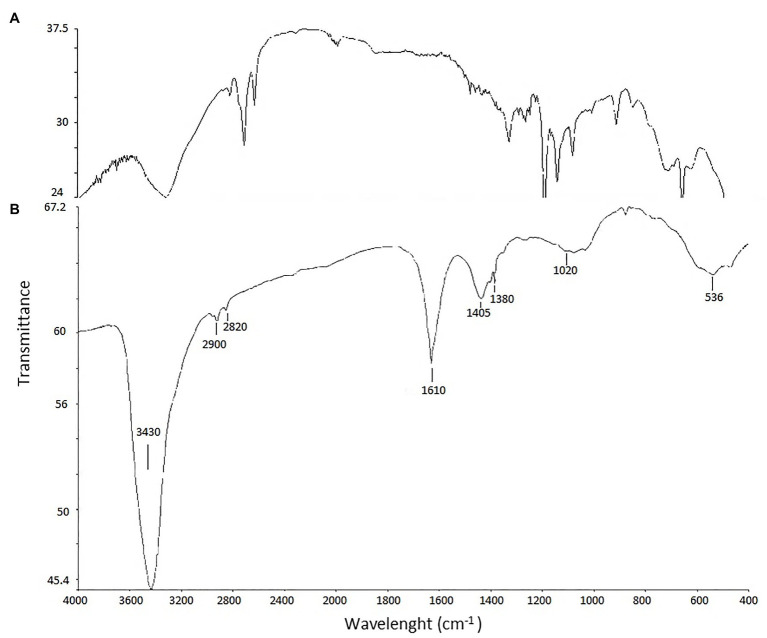
FTIR spectrum of biosynthesized AgNPs. AgNPs **(A)** and control **(B)**.

Fourier transform infrared analysis of biosynthesized AgNPs revealed presence of eight bands, namely at 3,430, 2,900, 2,820, 1,610, 1,405, 1,380, 1,020, and 536 cm^−1^ (Figure 5).

### MICs and MBCs of AgNPs

The tested AgNPs exhibited antimicrobial activity against both Gram-positive and Gram-negative bacteria (Table 1). However, *S. aureus* was much less sensitive (MIC = 128 μg ml^−1^ and MBC = 256 μg ml^−1^) than Gram-negative bacteria of *E. coli*, *K. pneumoniae* and *P. aeruginosa* (MIC values of 8, 32, and 8 μg ml^−1^, respectively; MBC values 32, 64, and 64, respectively) to tested AgNPs from actinobacterial strain SF23.

**Table 1:** Minimum inhibitory concentrations (MICs) and minimum biocidal concentrations (MBCs) of AgNPs (μg ml^−1^).

Test bacteria	AgNPs (μg ml^−1^)	
	MIC	MBC
*Escherichia coli* ATCC 8739	8	32
*Klebsiella pneumoniae* ATCC 700603	32	64
*Pseudomonas aeruginosa* ATCC 10145	8	64
*Staphylococcus aureus* ATCC 6538	128	256

### Quantitative Determination of ATP in Bacterial Cells After Treatment With AgNPs

**Figure 6:**
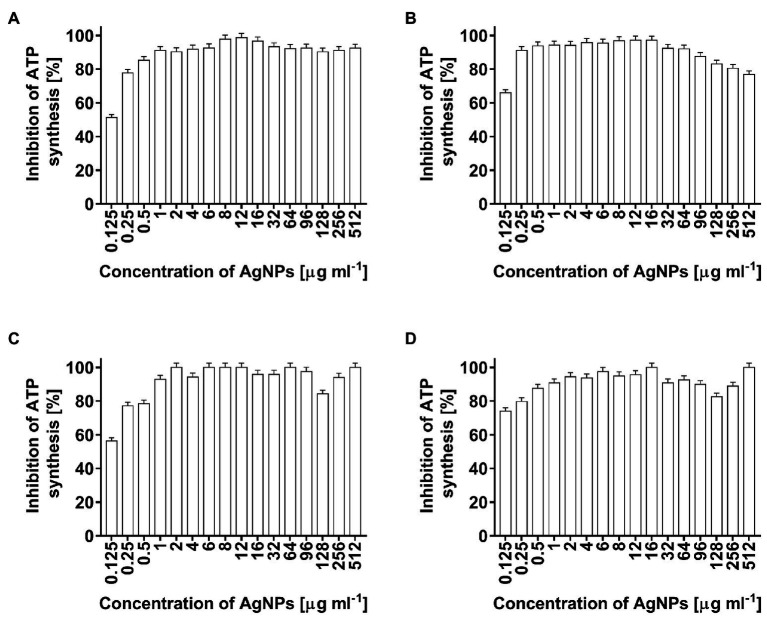
Inhibition of ATP synthesis (%) in *Escherichia coli* **(A)**, *Klebsiella pneumoniae* **(B)**, *Pseudomonas aeruginosa* **(C)**, and *Staphylococcus aureus* **(D)** cells after treatment with biosynthesized AgNPs at different concentrations (0.125–512 μg ml^−1^).

In the present study, the toxicity of AgNPs, as a potential antimicrobial agent, was estimated by the measurement of ATP levels in bacterial cells. The AgNP-treated cells of *E. coli*, *K. pneumoniae*, *P. aeruginosa*, and *S. aureus* showed significantly decreased ATP levels (Figures 6A–D, respectively). ATP synthesis in bacterial cells after treatment with the lowest used AgNPs concentration of 0.125 μg ml^−1^ decreased from 52% (*E. coli*) to 78% (*S. aureus*). At higher nanoparticle concentrations, ATP synthesis in bacterial cells was inhibited in a range of 76.8–100% (Figures 6A–D).

### Cytotoxic Effect of Silver Nanoparticles on MCF-7 Cancer Cells and RAW 264.7 Macrophages

**Figure 7:**
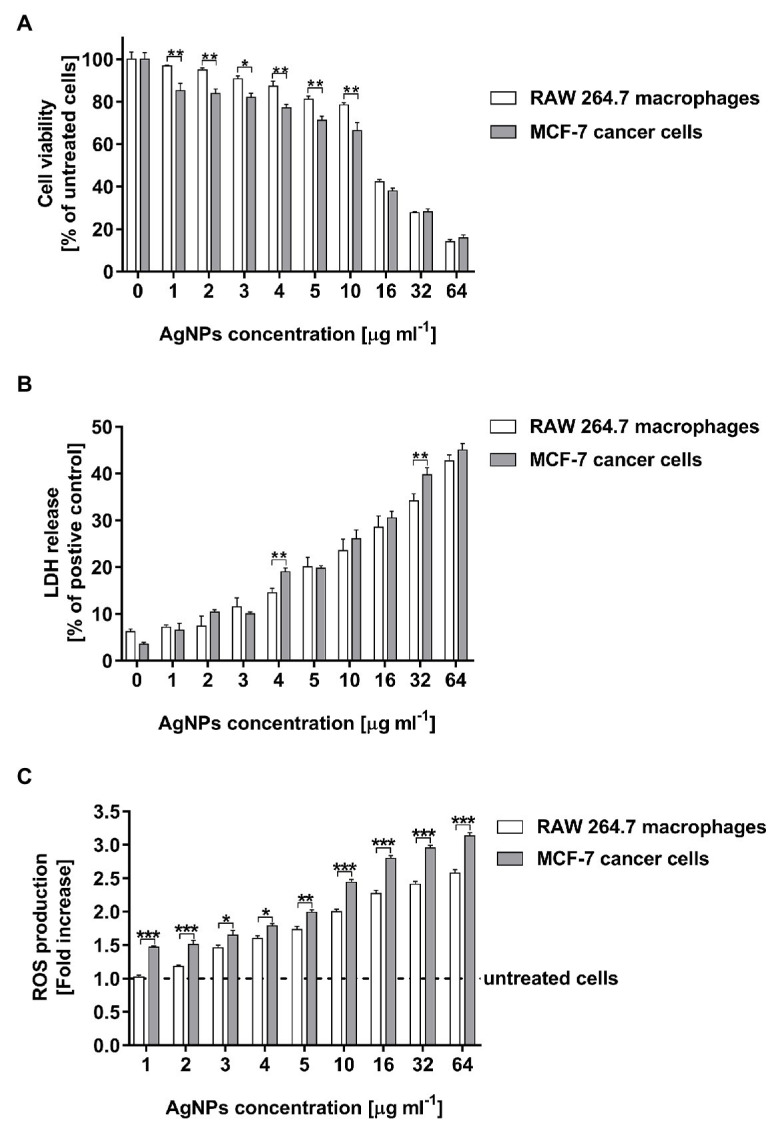
Cytotoxic activity of biosynthesized AgNPs toward human breast cancer cell line (MCF-7) and murine macrophage cell line (RAW 264.7) estimated by MTT assay **(A)**, LDH release **(B)**, and ROS production level **(C)**. Asterisks indicate significant differences between the viability of cells, the levels of LDH released from cells, or the ROS production in cells of both cell lines (***p < 0.001; **p < 0.01; *p < 0.1).

The cell viability of both tested cell lines were remarkably inhibited in the presence of AgNPs at a concentration of 16 μg ml^−1^ or higher (Figure 7A). AgNP concentration in a range of 16–64 μg ml^−1^ decreased RAW 264.7 cell viability from 42.2 ± 1.2 to 14.2 ± 1.0%, whereas in the case of MCF-7 cells the viability of measured cells was from 38.0 ± 1.3 to 15.8 ± 1.5%. Moreover, the decrease in RAW 264.7 cells viability was observed from a concentration of 4 μg ml^−1^ (*p* < 0.01), whereas in the case of MCF-7 cells the all tested doses of AgNPs significantly inhibited cell viability (*p* < 0.001).

The results of the MTT test were supported by the findings from the LDH assay. Total cytotoxicity was calculated by comparing the levels of LDH released from the AgNP-stimulated cells with the total levels of cellular LDH obtained by lysis of 1 × 10^4^ corresponding cells treated with 0.8% Triton X-100 solution. As compared with the control untreated cells, both tested cell lines stimulated with AgNPs exhibited an increase in LDH leakage in a dose-dependent manner. The LDH release significantly increased after treatment with AgNP concentrations in a range of 4–64 μg ml^−1^ (Figure 7B). The cytotoxicity measured for macrophages was from 14.6 ± 0.9 to 42.7 ± 1.3%, whereas for cancer cells was from 19.0 ± 0.8 to 45.0 ± 1.4%.

The cytotoxic effect of AgNPs was also expressed as the half-maximal inhibitory concentration (IC_50_) using data from the MTT test and LDH assay (Table 2). The IC_50_ growth inhibition values calculated for the MTT results against RAW 264.7 and MCF-7 cells were 16.3 ± 0.04 and 12.9 ± 0.05 μg ml^−1^, respectively. The IC_50_ values from LDH assay were found to be higher than those received from the MTT test (102.3 ± 0.07 and 76.2 ± 0.08 μg ml^−1^ for RAW 264.7 and MCF-7 cells, respectively). Importantly, the comparison of all IC_50_ results clearly indicated that the tested AgNPs demonstrated higher cytotoxicity against MCF-7 cancer cells in comparison with RAW 264.7 macrophages in terms of metabolic activity and membrane integrity.

**Table 2:** IC_50_ values (μg ml^−1^) obtained by MTT and LDH assay.

Cell line	MTT	LDH
**RAW 264.7**	16.3 ± 0.04	102.3 ± 0.07
**MCF-7**	12.9 ± 0.05	76.2 ± 0.08

The capacity of AgNPs to induce intracellular ROS production was assessed using 2',7'-dichlorofluorescein diacetate (DCFH-DA). The tested AgNPs stimulated ROS generation in RAW 264.7 macrophages as wells as in MCF-7 cancer cells in a dose-dependent manner. The increase in the ROS production in MCF-7 cells was noticed for all tested AgNP concentrations, whereas in the case of RAW 264.7 macrophages was observed from a concentration of 3 μg ml^−1^ (Figure 7C). Moreover, MCF-7 cells released a greater amount of ROS (from 1.47 ± 0.02 to 3.13 ± 0.05 fold increase) than RAW 264.7 macrophages (from 1.02 ± 0.03 to 2.58 ± 0.05 fold increase) during stimulation with the all tested concentrations of AgNPs (in a range from 1 to 64 μg ml^−1^).

### SDS-PAGE Protein Separation and Their LC-MS/MS Identification

**Figure 8:**
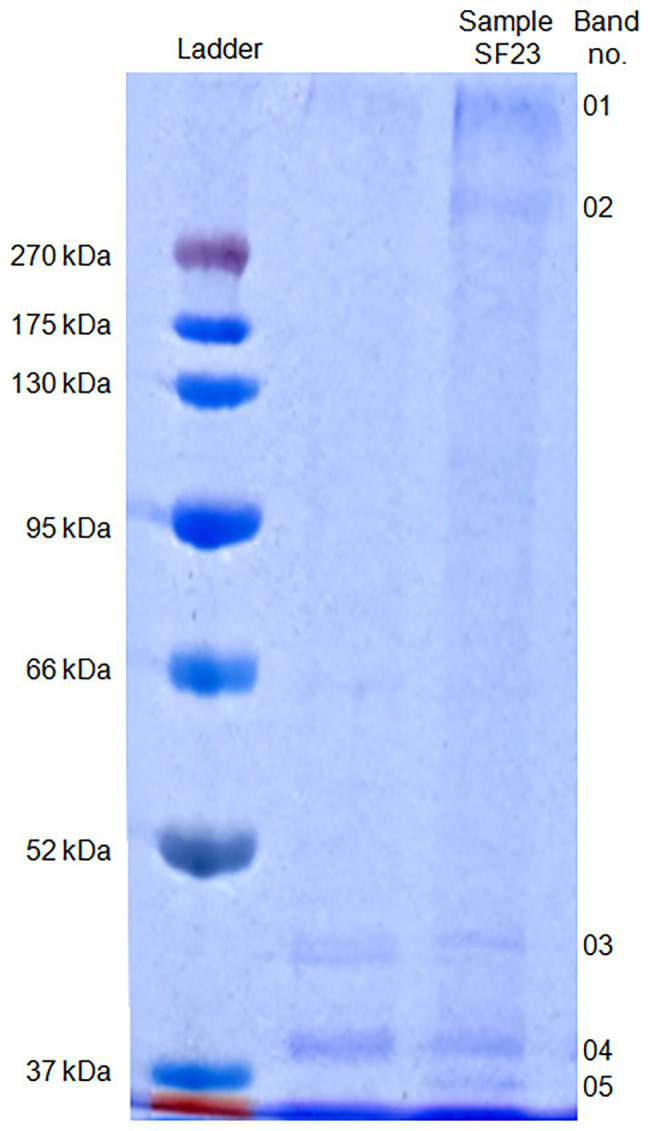
SDS-PAGE of proteins associated with biosynthesized AgNPs.

The SDS-PAGE electrophoresis of cell-free supernatant treated with AgNO_3_ showed the presence of five bands (01–05) which were absent in the control sample that was cell-free supernatant untreated with silver ions (Figure 8).

The molecular mass of separated proteins was calculated as follows: 280.9, 236.2, 38.2, 34.1, and 31.7 kDa, respectively. The peptides obtained after trypsin digestion and analyzed using LC-MS/MS were identified based on m/z (mass-to-charge ratio) value (Table 3). The excised band 01 was identified as a peptide with the highest homology to hypothetical protein from *E. coli* with an isoelectric point (pI) equal to 8.47 and molecular mass 174.0 kDa. Peptide 02 was found to have the highest homology to translation elongation factor Tu (cover 48%) from *Delftia* sp. 68-7 with pI = 5.20 and unknown molecular mass and to a hypothetical protein from *E. coli* (cover 47%) with pI = 8.47 and a molecular mass of 151.0 kDa. The excised bands 03, 04, and 05 were peptides showing the highest similarity to *Pseudomonas rhodesiae* OprD family porin (53% cover), *Cupravidus* sp. porin (60% cover), and *Delftia* sp. porin (48% cover) with unknown molecular mass (all) and pI equal to 5.73, 8.81, and 9.11, respectively (Table 3).

**Table 3:** Identification of proteins associated with silver nanoparticles synthesized from actinobacterial strain SF23.

Band number	Accession	Description	Taxonomy	Coverage (%)	Molecular weight (kDa)	Calculated pI	Score
**01**	WP_108997678.1	Hypothetical protein, partial	*Escherichia coli*	38.0	174.0	8.47	2,932
	WP_016451823.1	MULTISPECIES: hypothetical protein	*Delftia*	30.0	Unknown	5.89	2,110
**02**	WP_108997678.1	Hypothetical protein, partial	*Escherichia coli*	47.0	151.0	8.47	3,665
	WP_016451823.1	MULTISPECIES: hypothetical protein	*Delftia*	40.0	Unknown	5.89	3,125
	OJX13945.1	Translation elongation factor Tu, partial	*Delftia* sp. 67-8	48.0	Unknown	5.20	1,239
**03**	WP_108997678.1	Hypothetical protein, partial	*Escherichia coli*	41.0	44.0	8.47	2,589
	WP_040269305.1	OprD family porin	*Pseudomonas rhodesiae*	53.0	Unknown	5.73	1,748
	WP_090400570.1	OprD family porin	*Pseudomonas grimontii*	37.0	Unknown	5.43	1,203
**04**	WP_108997678.1	Hypothetical protein, partial	*Escherichia coli*	41.0	38.0	8.47	3,391
	WP_008643071.1	MULTISPECIES: porin	*Cupriavidus*	60.0	Unknown	8.81	3,152
	WP_008645123.1	Porin	*Cupriavidus* sp. HMR-1	40.0	Unknown	9.08	1,750
	WP_016448722.1	MULTISPECIES: porin	*Delftia*	45.0	Unknown	9.11	1,254
**05**	WP_108997678.1	Hypothetical protein, partial	*Escherichia coli*	38.0	36.0	8.47	2,182
	WP_016448722.1	MULTISPECIES: porin	*Delftia*	48.0	Unknown	9.11	1,985
	OJX13945.1	Translation elongation factor Tu, partial	*Delftia* sp. 67-8	43.0	Unknown	5.20	1,111
	WP_007970421.1	MULTISPECIES: elongation factor Tu	*Pseudomonas*	40.0	Unknown	5.23	1,002

## Discussion

The type, morphology, stability, and aggregation of nanoparticles are the main properties determining their biological activity including the toxic effect on biological systems (Jeevanandam et al., 2018; Auría-Soro et al., 2019). In the present study, analyses of the physical properties of biogenic AgNPs confirmed their crystalline nature (Oves et al., 2019), revealed their small size (mean size = 13.2 nm), good stability, and coating with biological functional groups. The size of nanomaterials is an important feature that affects their physical properties, penetration into cells, and interactions with cell molecules. Smaller nanoparticles have a relatively larger surface area when compared to the same volume of material made up of bigger particles thus such nanoparticles have higher surface activity (Navya and Daima, 2016). The smaller nanoparticles, the easier penetrate through biological membranes (Jeevanandam et al., 2018). They can also easily pass through the blood-brain barrier if their diameter is under 12 nm (Oberdörster et al., 2004; Sarin et al., 2008; Sonavane et al., 2008). Moreover, the nanoparticles also cause a dose-dependent increase in oxidation and DNA damage (Donaldson and Stone, 2003). The stability of nanoparticles resulting from the electrostatic repulsion between particles in the solution, prevent their agglomeration and is related to zeta potential value. Generally, surface charge of nanoparticles is related to their cytotoxicity. This toxic effect toward cells increases with an increase in the nanoparticle surface charge. Therefore, higher positively charged nanoparticles stronger interact with negatively charged cell surface thus more easily penetrate into cells and consequently may generate higher cytotoxic effect than negatively charged ones (Foroozandeh and Aziz, 2018; Auría-Soro et al., 2019). In contrast, the closer the zeta potential value to −30 mV, the more stable are metal nanoparticles and their harmful effect to biological systems is lower (Gade et al., 2010; Rezvani Amin et al., 2019). Interestingly, positively charged nanoparticles tend to accumulate more in tumor cells than these with negative surface charge (Hoshyar et al., 2016). Both, small in size (1.7–50 nm) and negatively charged AgNPs with moderate and good stability (zeta potential from −14.7 to −18.0 mV) as well as high stability (−35.3 to −81.5 mV) synthesized by filamentous actinobacteria have been reported by many authors (Prakasham et al., 2014; Rathod et al., 2016; Wypij et al., 2018b; Bakhtiari-Sardari et al., 2020). Therefore, our findings are in line with these previously published. However, differences in the nanoparticle size and charge might be related to strain specificity, growth conditions of strain, and conditions used during the synthesis process (Kupryashina et al., 2013; Mohanta and Behera, 2014; Rai et al., 2016). Some subtle differences have been observed even in the same isolate (Wypij et al., 2017, 2020). The FTIR bands observed in region 3,430 cm^−1^ is characteristic for vibrations of hydroxyl and amino groups (Shanmugaiah et al., 2015; Qayyum et al., 2017; Wypij et al., 2018a; Oves et al., 2019). The peak at 2,900 cm^−1^ can be assigned to the C–H-asym. stretching vibration (Karthik et al., 2013; Qayyum et al., 2017), and peak at 2,820 cm^−1^ can be associated with -CH_3_-sym. stretching vibration (Rai et al., 2015). The strong band at 1,610 cm^−1^ be assigned to stretching of carbonyl groups (C=O) (Zaki et al., 2011; Oves et al., 2019) whereas bands in spectrum 1,380–1,405 cm^−1^ correspond to C–N stretching and NH bending, indicating the presence of aliphatic groups of amide II (Zaki et al., 2011). The peak at 1,020 cm^−1^can be assigned to C–O stretching vibration whereas this one at 536 cm^−1^ corresponds to C–Cl stretching in the alkyl group (Gurunathan et al., 2015). Based on obtained results it was concluded that AgNPs synthesized from actinobacterial strain SF23 were capped with molecules with amino bonds. It is claimed that such capping agents are responsible for the reduction of Ag ions to AgNPs and stability of formed nanostructures (Sanghi and Verma, 2009; Manivasagan et al., 2013; Qayyum et al., 2017; Oves et al., 2019).

Although the antibacterial mechanisms of AgNP action have been studied and discussed extensively, they have not yet been fully elucidated. Two main antimicrobial mechanisms of AgNPs are widely accepted, namely direct and ion-mediated destructions (Qing et al., 2018).

It was also reported that the activity of AgNPs against Gram-negative bacteria was stronger than Gram-positive bacteria (Rathod et al., 2016; Qing et al., 2018; Devagi et al., 2020) which was confirmed by results of the present study. This phenomenon has been explained by the existing difference in the cell wall structure between Gram-positive bacteria, which is mainly composed of thick layer of peptidoglycan, and Gram-negative bacteria (Chatterjee et al., 2015; Qing et al., 2018). However, some reports contradict this finding (Prakasham et al., 2014; Wypij et al., 2017; Dayma et al., 2019; Bakhtiari-Sardari et al., 2020) or show variable susceptibility within these bacterial groups (Składanowski et al., 2016; Wypij et al., 2018a,b; Hossain et al., 2019).

Exposure of microorganisms to AgNPs leads to adhesion of nanoparticles on the surface of the cell wall (Abbaszadegan et al., 2015). AgNPs interact with the sulfur-containing proteins present in the cell wall leading to irreversible changes in its structure thus its weakening (Ghosh et al., 2012). Consequently, AgNPs affects the integrity of lipid bilayer and permeability of the cell membrane which is responsible for the proper regulation of transport through the plasma (Panacek et al., 2006; Ghosh et al., 2012; Chauhan et al., 2013). Furthermore, the antibacterial mechanism of AgNPs is associated with the generation of high level of ROS and free radical species (e.g., hydrogen peroxide, superoxide anion, hydroxyl radical, hypochlorous acid, and singlet oxygen) (Siritongsuk et al., 2016; Gomaa, 2017) which inhibit respiration and growth of cells (Su et al., 2009; Quinteros et al., 2016). Increased level of ROS lead to an apoptosis-like response, lipid peroxidation, depletion of antioxidant enzyme such as GSH, and DNA damage (Lee et al., 2014; Korshed et al., 2016). Moreover, silver ions that are released from nanoparticles contribute to their biocidal properties (Morones et al., 2005; Manivasagan et al., 2013). They can also affect membrane transport and the release of potassium ions from the microbial cells. Consequently, the increased membrane permeability leads to leakage of cellular contents, including ions, proteins, reducing sugars, and adenosine triphosphate (ATP), cellular energy reservoir (Lok et al., 2006; Kim et al., 2011; Chauhan et al., 2013; Li et al., 2013). AgNPs and/or silver ions inside the microbial cells can interact with cellular structures (e.g., ribosomes) and biomolecules such as proteins, lipids, and DNA, which have a damaging effect on microbial cells. They disturb DNA replication, translation process in ribosomes, and protein activities (Morones et al., 2005; Jung et al., 2008; Rai et al., 2012; Chauhan et al., 2013).

Adenosine triphosphate is an essential metabolite that plays fundamental roles as an energy transfer and major signaling molecule (Mempin et al., 2013). Overall, AgNPs from actinobacterial strain SF23 significantly inhibited ATP synthesis in test Gram-negative and Gram-positive bacteria. This confirms that inhibition of ATP synthesis can be one of the antimicrobial mechanism of AgNPs. Recently, Yuan et al. (2017) reported similar findings and found that AgNPs synthesized using bio-molecule called quercetin significantly decreased ATP synthesis in cells of *P. aeruginosa* and *S. aureus* (about 3.5 times less) when compared to untreated cells. Cellular stress generated in *P. aeruginosa* and *S. aureus* after treatment with AgNPs significantly negatively affected ATP synthesis and consequently bacterial growth and reproduction (Yuan et al., 2017). Moreover, it was also found that AgNPs affected FOF1-ATPase activity and H^+^-coupled transport in the bacterial cell membrane (Vardanyan et al., 2015). The H^+^ and K^+^ transport was impaired after exposure bacterial cells to AgNPs and even if N, N0-dicyclohexylcarbodiimide, an inhibitor of FOF1, was present. These findings revealed that this membrane ATPase is a potential target for AgNPs and its inhibition may affects metabolic activity in bacterial cells (Vardanyan et al., 2015; Yuan et al., 2017). This high toxicity of AgNPs toward microbial cell showed their potential as an antimicrobial agent.

The use of AgNPs for biomedical purposes, especially *in vivo* applications, requires determination of their cytotoxic effect (Lewinski et al., 2008). Although many studies on the toxicity of biogenic AgNPs in various cell lines have been reported (Gurunathan et al., 2013, 2018; Gopinath et al., 2017; Wypij et al., 2018a; Hamed et al., 2020), the mechanisms underlying the toxicity in eukaryotic cells are still unclear.

Similarly, to prokaryotic cells, in eukaryotic ones, the size of AgNPs is a key factor determining their penetration into the cells by diffusion (translocation), endocytosis, or phagocytosis (Zhang et al., 2015). Nanoparticles present in cells may accumulate in the mitochondria causing reduction in mitochondrial membrane potential that interrupts ATP synthesis and leading to ROS formation (Rani et al., 2009; Grzelak et al., 2018). Not only AgNPs themselves, but also released silver ions generate ROS in cells. The high ROS amount leads to oxidative stress responsible for damage of cell membrane, which can be observed as LDH release, and intracellular proteins, lipids, and DNA (Riaz Ahmed et al., 2017; Liao et al., 2019). Higher ROS level induces cell respond by activating pro-inflammatory signaling cascades, and finally programmed cell death by either apoptosis or necrosis (Finkiel and Holbrook, 2000; Martindale and Holbrook, 2002; Stensberg et al., 2011; Lee et al., 2013; Akter et al., 2018). ROS at low-level function as signaling molecules in cells which regulates various cellular functions. In addition, silver ions released from AgNPs may increase their cytotoxic effect by inducing cascades that lead to intracellular toxicity defined as the “lysosome-enhanced Trojan horse effect” (Sabella et al., 2014). Moreover, AgNPs can interact with the membrane proteins and activate signaling pathways leading to the inhibition of cell proliferation (Riaz Ahmed et al., 2017).

In the present study, the cytotoxicity of AgNPs was evaluated using two different assays. In MTT assay, formazan accumulation directly reflects the mitochondrial activity in the live cells, which is an indirect measurement for the cell viability. On the other hand, the cell membrane damaged by the cytotoxic agent such as AgNPs allows the release of intracellular LDH molecules into the culture medium. Therefore, the LDH leakage indirectly reflects the compromised cell membrane integrity, which is associated with necrosis (Kwan et al., 2016). The IC_50_ values clearly demonstrated that AgNPs were more cytotoxic against MCF-7 cells in comparison with RAW 264.7 macrophages. Similarly, Pandian et al. (2019) showed that the biosynthesized PEGylated AgNPs were less cytotoxic to RAW 264.7 macrophages compared to human cervical carcinoma HeLa cells. Therefore, our findings seem to be promising since the use of biosynthesized AgNPs is considered as an effective anticancer therapy (Firdhouse and Lalitha, 2013; Gajendran et al., 2014) while activation of macrophages can be involved in defense mechanism against tumors through lysis of cancer cells by liberating oxygen radicals and tumor necrosis factors (Klimp et al., 2002) or by controlling cancer through innate immunity, namely by stimulating the functions of T and B cells (Mills et al., 2016). Importantly, AgNPs stimulated MCF-7 cells to release a greater amount of ROS than RAW 264.7 macrophages, which correlates with higher cancer cell mortality. Therefore, we presume that AgNP-induced cell death can be partially mediated by ROS production.

It is well-known that despite potent antibacterial activity of AgNPs and wide range of biomedical applications, their use as therapeutic agents is limited because of their cytotoxicity against mammalian cells (Jena et al., 2012). According to the ISO 10993-1 (2018; Biological evaluation of medical devices: Part 1: evaluation and testing within a risk management process), if the cell viability was reduced to <70% of the blank, it would have a cytotoxicity potential. In our study, the viability of RAW 264.7 cells was higher than 70% when the cells were stimulated with AgNPs at a concentration up to 10 μg ml^−1^ (78.4 ± 1.1%) and the values of IC_50_ obtained by the MTT assay was 16.3 ± 0.04 μg ml^−1^. At the same time, among the tested bacteria, MICs values were lower only for Gram-negative bacteria, such as *E. coli* and *P. aeruginosa* (MIC = 8 μg ml^−1^) and higher for Gram-positive bacteria. There are some evidences indicating that macrophage cell lines, such as RAW 264.7 and J774.1 cells show high sensitivity to AgNPs stimulation due to the activation of scavenger receptor pathway and the scavenger function of macrophages (Singh and Ramarao, 2012; Dalzon et al., 2017). Moreover, the increased cytotoxicity of AgNPs in macrophages can be induced by phagocytosis of AgNPs that contribute to the ROS generation, and as a consequence, to the stimulation of tumor necrosis factor *α* (TNF-α) production. This cytokine is a well-known factor, which causes the damage of cell membrane and apoptosis of cells, including macrophages (Park et al., 2010). Based on these findings, we are aware that there is a need to search such concentrations of the tested AgNPs, which will be microbicidal and not cytotoxic against immune cells, such as macrophage at the same time. Our results show that the tested AgNPs can be considered as a potential antimicrobial agent, which exhibits antibacterial activity against Gram-negative bacteria at a doses of being safe for macrophages.

As mentioned previously, the proteins of natural origin are considered as both reducing and capping agents of biosynthesized nanoparticles (Akhtar et al., 2013; Karatoprak et al., 2017; Siddiqi et al., 2018). Recently, Shu et al. (2020) used yeast extract for the synthesis of AgNPs and reported that amino acids, alpha-linolenic acid, and aminobutyric acid are responsible for reducing of silver ions and capping of AgNPs. The authors found that due to capping by biomolecules, the stability of monodispersed AgNPs perpetuate for more than 1 year. These capping agents protect AgNPs from aggregation as well as from oxidation of Ag^0^ to Ag+ ions. Moreover, the protein coating of AgNPs can be used for formation of drug delivery systems for the human cells by attaching different molecules, including drugs and nucleic acids (Rodriguez et al., 2013).

However, available reports on capping agents of biogenic nanoparticles, especially ones of the bacterial origin, show that the current knowledge on this subject is still in its infancy. Chowdhury et al. (2014) reported the presence of several protein bands at molecular weights between 50 and 116 kDa after SDS-PAGE of AgNPs synthesized using a cell-free extract of phytopathogenic soil-borne fungus *Macrophomina phaseolina* (Tassi) Goid. However, these proteins were not identified by the authors (Chowdhury et al., 2014). Ballottin et al. (2016) identified the extracellular protein secreted by an endophytic *Aspergillus tubingensis* that was responsible for capping and stabilization of biosynthesized AgNPs. This protein was covalently bonded to AgNPs by functional groups of amino acids, mainly thiol group (HS–) of cysteine and less often by amino groups (H_2_N–) forming S-Ag and N–Ag bonds, respectively. Further studies revealed the presence of other proteins of an enzymatic nature, namely acid phosphatase (EC 3.1.3.2), glucoamylase (1,4-α-D-glucanglucohydrolase, EC 3.2.1.3), glucanosyltransferase (EC 2.4), and serine carboxypeptidase (EC 3.4.21.26), which were associated with AgNPs by electrostatic and additional protein-protein interactions and responsible for the metabolic activities of *A. tubingensis* (Ballottin et al., 2016). More recently, Guilger-Casagrande et al. (2021) studied the significance of capping agents of AgNPs synthesized from *Trichoderma harzianum* and compared with AgNPs without caps. Interestingly, the capped AgNPs demonstrated remarkable inhibition of *Sclerotinia sclerotiorum*, a plant pathogen. Consequently, they put forth a hypothesis that nanoparticles and capping agent (active hydrolytic enzymes of *T. harzianum*) act synergistically against *S. sclerotiorum*. It is already known that hydrolytic enzymes secreted by *T. harzianum* play an important role in cell wall degradation of plant pathogens (Qualhato et al., 2013). The AgNPs synthesized by peel extract of *Citrus sinensis* were capped by three different proteins with aspartic-type endopeptidase, GSH *S*-transferase, and oxalate oxidase activities (Barros et al., 2018). Recently, Wypij et al. (2020) separated proteins associated with AgNPs from actinobacteria using SDS-PAGE and found three protein bands that showed the highest homology to porins previously described in Gram-negative bacteria. Similarly, in the present work SDS-PAGE analysis showed the presence of five proteins bands at a molecular weight between 31.7 and 280.9 kDa. These proteins showed the highest homology to hypothetical proteins and porins from Gram-negative bacteria such as *E. coli*, *Delftia* sp. and *Pseudomonas rhodesiae*.

The binding of proteins on a nanoparticle surface depends on surface charge. Proteins associated with AgNPs change dramatically their physical and chemical properties, mainly surface charge and composition. The higher surface energy the better binding of protein with nanoparticles. However, the protein adsorption is also regulated by protein-protein interactions (Nguyen and Lee, 2017). Although the capping proteins can increase internalization of AgNPs into cells *via* endocytosis, regulate cellular uptake and bioavailability of AgNPs, they can also be responsible for the toxic activity of AgNPs (Ritz et al., 2015; Barbalinardo et al., 2018). Therefore, further studies are needed to understand the type of proteins and their precise role in formation and bioactivity of actinobacterial-mediated nanoparticles.

To sum up, actinobacterial-mediated AgNPs were characterized and analyzed for their antibacterial and cytotoxic activities. In addition, their capping proteins were studied. These biogenic nanoparticles showed good stability, high antibacterial activity, and inhibited ATP synthesis as a potential antimicrobial mechanism of action. They showed cytotoxicity against breast cancer cells and macrophages but higher toward the former ones. Based on our findings, it can be concluded that biosynthesized AgNPs from actinobacterial strain SF23 are capped with proteins and may be a potential cytotoxic agent against cancer cells and bacteria. In addition, the presence of nanoparticle coating with many functional groups give opportunities for further modifications to improve their biological activity, namely antimicrobial and anticancer ones, and biocompatibility. Considering the potential of these nanoparticles, they can be recommended for biomedical applications after further *in vivo* studies.

## Data Availability Statement

The original contributions presented in the study are included in the article/supplementary material, further inquiries can be directed to the corresponding authors.

## Author Contributions

MW and PG conceptualized the study. MW was responsible for synthesis of AgNPs, UV-Vis, TEM, XRD, and FTIR analyses. MR was responsible for the Zeta potential analyses. JT-W, MW, and PG carried out analyses of ATP synthesis and MIC and MBC assays after treatment with AgNPs. MW and TJ performed cytotoxicity analyses of AgNPs toward cell lines. MW and MO performed analyses of capping proteins. MW, TJ, and JT-W were responsible for the original draft preparation. PG supervised the study. PG and MR reviewed and edited the manuscript. MW acquired funds for studies. All authors contributed to the article and approved the submitted version.

### Conflict of Interest

The authors declare that the research was conducted in the absence of any commercial or financial relationships that could be construed as a potential conflict of interest.

## Footnotes

^1^https://www.matrixscience.com
